# Bisphenol A exposure affects specific gut taxa and drives microbiota dynamics in childhood obesity

**DOI:** 10.1128/msystems.00957-23

**Published:** 2024-03-01

**Authors:** Ana Lopez-Moreno, Klara Cerk, Lourdes Rodrigo, Antonio Suarez, Margarita Aguilera, Alicia Ruiz-Rodriguez

**Affiliations:** 1 Department of Microbiology, Faculty of Pharmacy, University of Granada, Campus of Cartuja, Granada, Spain; 2 Institute of Nutrition and Food Technology "José Mataix" (INYTA), Centre of Biomedical Research, University of Granada, Granada, Spain; ^3^Instituto de Investigación Biosanitaria ibs, Granada, Spain; 4 Quadram Institute Bioscience, Rosalind Franklin Road, Norwich Research Park, Norwich, United Kingdom; 5 Department of Biochemistry and Molecular Biology II, Faculty of Pharmacy, Campus of Cartuja, University of Granada, Granada, Spain; Génomique Métabolique, Genoscope, Institut François Jacob, CEA, CNRS, Université Évry, Université Paris-Saclay, Evry, France

**Keywords:** xenobiotics, BPA, obesity, microbiota, culturomics, amplicon-sequencing

## Abstract

Cumulative xenobiotic exposure has an environmental and human health impact which is currently assessed under the One Health approach. Bisphenol A (BPA) exposure and its potential link with childhood obesity that has parallelly increased during the last decades deserve special attention. It stands during prenatal or early life and could trigger comorbidities and non-communicable diseases along life. Accumulation in the nature of synthetic chemicals supports the “environmental obesogen” hypothesis, such as BPA. This estrogen-mimicking xenobiotic has shown endocrine disruptive and obesogenic effects accompanied by gut microbiota misbalance that is not yet well elucidated. This study aimed to investigate specific microbiota taxa isolated and selected by direct BPA exposure and reveal its role on the overall children microbiota community and dynamics, driving toward specific obesity dysbiosis. A total of 333 BPA-resistant isolated species obtained through culturing after several exposure conditions were evaluated for their role and interplay with the global microbial community. The selected BPA-cultured taxa biomarkers showed a significant impact on alpha diversity. Specifically, *Clostridium* and *Romboutsia* were positively associated promoting the richness of microbiota communities, while *Intestinibacter*, *Escherichia-Shigella*, *Bifidobacterium*, and *Lactobacillus* were negatively associated. Microbial community dynamics and networks analyses showed differences according to the study groups. The normal-weight children group exhibited a more enriched, structured, and connected taxa network compared to overweight and obese groups, which could represent a more resilient community to xenobiotic substances. In this sense, subnetwork analysis generated with the BPA-cultured genera showed a correlation between taxa connectivity and more diverse potential enzymatic BPA degradation capacities.

**IMPORTANCE:**

Our findings indicate how gut microbiota taxa with the capacity to grow in BPA were differentially represented within differential body mass index children study groups and how these taxa affected the overall dynamics toward patterns of diversity generally recognized in dysbiosis. Community network and subnetwork analyses corroborated the better connectedness and stability profiles for normal-weight group compared to the overweight and obese groups.

## INTRODUCTION

Cumulative environmental chemical exposures during early life are critical for lifelong health, with environmental factors estimated to contribute to 70%–90% of disease risk (1). The exposome, introduced over a decade ago, encompasses all environmental factors affecting an individual’s life, making it difficult to accurately be biomonitored (2, 3). The impact of exposome in early life development, including the fetal and childhood stages, might have significant implications for chronic diseases in adulthood (1, 4). In this sense, a fundamental principle in biology is that the phenotype is determined by the permanent interplay between the individual genetics and the environment throughout one’s lifetime (5).

Importantly, environment pollutants seem to be linked to the increased incidence of metabolic disorders that has become a global health issue over the past decades (6). Especially worrying is the recent trend of increasing worldwide prevalence of childhood obesity, which may result in metabolic disorders during adulthood (7, 8). Obesity is associated with many comorbidities and chronic conditions, including type 2 diabetes mellitus, high blood pressure, cardiovascular disease, cancer progression, and non-alcoholic fatty liver disease (9–11). While excessive caloric intake and a sedentary lifestyle are the main factors, human data integration, including the microbiome interactions, its function and metabolites have widely demonstrated to be decisive for determining obesogenic phenotypes (12). Moreover, recent evidence suggests that the exposure to xenobiotic chemicals that disrupt adipogenesis and energy balance may also play a key role (13, 14). The rise in obesity rates has been linked to the increase in synthetic chemical production, which supports the “environmental obesogen” hypothesis (4).

Special attention should be paid to the cumulative exposure to endocrine disrupting compounds that have shown obesogenic effects during early life (15). Bisphenols are estrogen-mimicking chemicals present in manufacturing polycarbonate plastics and epoxy resins as well as in thermal printing papers (16). Moreover, bisphenol A (BPA) has been detected in human serum, urine, saliva, hair, tissue, and blood, indicating its accumulative presence and systemic risks (17, 18). Differential individual effects have been observed in relation to BPA, and it seems that the gut microbiota could be a key player in the individual response to the exposure. Multiple studies have confirmed that BPA exposure can affect the microbiome, sometimes increasing microbial biomarkers of dysbiosis, such as the increase of the *Pseudomonadota* members (19). Our understanding of the cause-and-effect relationship in host-microbiome interactions is still limited; however, there are some indications that the enzymes responsible for degrading most plastics and BPA were those produced by gut-associated microbial taxa, rather than those produced by the host (20).

Nowadays, there are multiple methods for studying the gut microbiota, each of them with their own advantages and limitations. Specifically in this field, microbial culturing approaches are needed for obtaining microorganisms and their enzymes involved in BPA degradation and for exploring their potential as beneficial microbes and as next-generation probiotics in xenobiotic-triggered dysbiosis (21, 22). Importantly, culturomics can also detect minority taxa that may be underrepresented in metagenomic analysis, complementing and filling gaps in identifying non-cultivable taxa (23, 24).

This study aimed to investigate the impact of BPA on gut microbiota taxa in relation to childhood obesity. We first isolated BPA-tolerant species and selected differential taxa biomarkers from the normal-weight (NW), overweight (OW), and obesity (O) groups. The interplay of these taxa biomarkers with the global community defined by 16S rRNA gene amplicon sequencing was further analyzed, exploring diversity indices, community structure, and ecological networks. Our findings pursued not only a better understanding of the relation between BPA, gut microbial dysbiosis, and obesogenic phenotypes but also the identification of potential taxa or consortia biomarkers associated with BPA exposure.

## RESULTS

### Characterization of BPA-tolerant gut microbiota isolates through culturing

A total of 333 BPA-tolerant isolates were obtained. Most of the selected colonies were isolated by exposing the samples to 20 (43.5%) and 50 (46.8%) ppm of BPA. The medium rendering the highest number of BPA-tolerant taxa counts was brain-heart infusion (BHI; 31.9%), followed by Man, Rogosa, and Sharpe (MRS; 21.6%); reinforced clostridial medium (RCM; 15.6%), Gifu anaerobic medium modified with agar (GAMa; 15.6%), and Gifu anaerobic medium modified with gellan gum (GAMg; 15.3%). According to the study group classification, 171 isolates were picked from the NW; 120, from the O; and 42, from the OW groups similarly distributed within 20 and 50 ppm of BPA.

**Fig 1:**
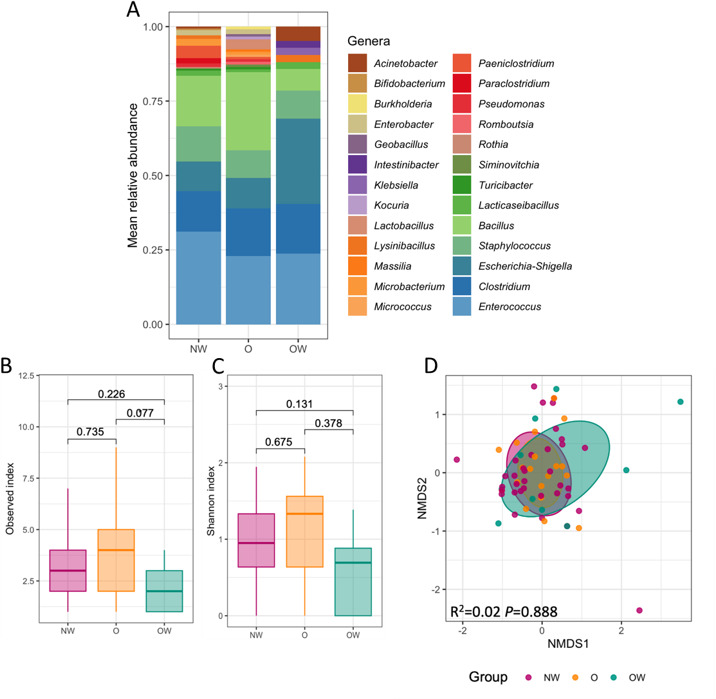
Description of the children’s gut microbiota in NW, OW, and O groups assessed by culturomics. (**A)** Cumulative bar plot showing the phylogeny of isolated microorganisms at the genus level. (**B and C)** Box plots of α-diversity indices of the microbiota across the three groups. Medians and interquartile ranges are shown. Group differences in the observed number of amplicon sequence variants and Shannon α-diversity were calculated using multivariable linear models on the log-transformed data and corrected for age. *P* values shown in the figure correspond to pairwise comparisons assessed with the multcomp package. (**D)** Nonmetric multidimensional scaling (NMDS) plot based on Bray-Curtis dissimilarity between samples, with data points and ellipses colored by study groups. Results of the permutational multivariate analysis of variance (PERMANOVA) test (*R*^2^ and *P* value corrected for age) based on Bray-Curtis dissimilarity index ordination are indicated within the plot. Ellipses represent the standard deviation of all points within a study group.

The bacterial species isolated in this study belonged to three different phyla, with the majority belonging to the *Bacillota* (79.9%), followed by *Pseudomonadota* (16.2%), and the remaining (2.7%) of the bacteria species belonging to *Actinomycetota*. From 26 genera identified, *Bacillus*, *Enterococcus*, *Escherichia-Shigella*, *Staphylococcus*, and *Clostridium* were the most representative isolates in NW, O, and OW groups (Fig. 1A). We observed different profiles between our study groups, especially when focusing on the minor genera (Fig. 1A). Species richness and Shannon’s diversity index values were not significantly different between NW and O groups, while a significant trend was observed when comparing OW with O and NW samples (Fig. 1B), likely due to the lower number of isolates retained from the OW group. β Diversity on Bray-Curtis dissimilarity metrics was not significantly different between the experimental groups (Fig. 1C).

**Fig 2:**
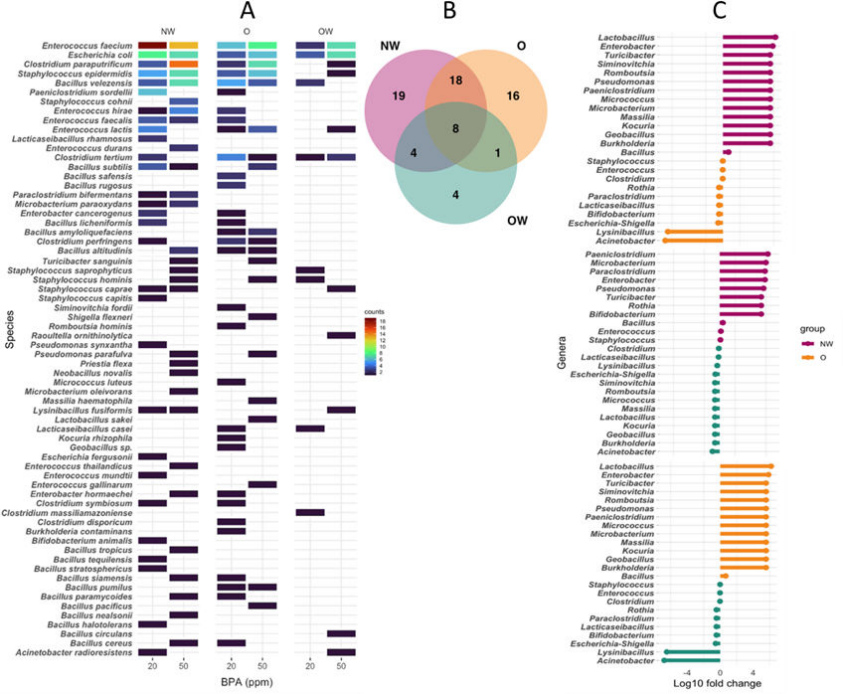
**(A)** Tile map showing frequency of species isolation at 20 and 50 ppm of BPA in NW, O, and OW groups. The species are ordered according to their counts. (**B)** Venn diagram analysis shows common, shared, and unique species in the study groups. (**C)** Fold change (log_10_) plots of BPA-tolerant genera (NW vs O; NW vs OW; O vs OW).

The 333 isolated bacteria were taxonomically assigned to 66 bacterial species. The most abundant species were *Enterococcus faecium* (17.4%), *Escherichia coli* (11.4%), *Clostridium paraputrificum* (9%), *Bacillus velezensis* (7.2%), and *Staphylococcus epidermidis* (6.6%) with tolerance to 20 and 50 ppm of BPA in the three groups (Fig. 2A). The species *Enterococcus faecium*, *Enterococcus lactis*, *Escherichia coli*, *Clostridium paraputrificum*, *Clostridium tertium*, *Bacillus velezensis*, *Staphylococcus epidermidis*, and *Staphylococcus hominis* were shared between the three study groups (NW, O, and OW) (Fig. 2B). Unique species were observed belonging to the groups, 19 in the NW group, 16 in the O group, and 4 in the OW group. NW and O groups shared the highest number of species (18), while NW and OW shared four and O and OW only one.

A log10 fold change calculation was performed based on the abundance of each genus in order to associate its presence to the study groups (Fig. 2C). Some groups’ signature genera were found, such as *Paeniclostridium*, *Microbacterium*, and *Turicibacter* that were consistently overrepresented in the NW group when compared with O and OW groups or *Acinetobacter* that was overrepresented in the OW group when compared to NW and O groups. Next, a similar analysis was performed at the species level to explore the differences between NW and O groups with the most distinct body mass index (BMI) values. Isolates assigned to *Lysinibacillus fusiformis*, *Staphylococcus caprae*, *Lacticaseibacillus rhamnosus*, *Enterococcus durans*, *Microbacterium paraoxydans*, *Paraclostridium bifermentans*, and *Staphylococcus cohnii* were overrepresented in NW samples, while *Lactobacillus sakei*, *Bacillus rugosus*, *Bacillus safensis*,and *Bacillus pumilus* were mostly isolated from the O samples.

### Characterization of gut microbiota communities through 16S rRNA gene amplicon-sequencing

**Fig 3:**
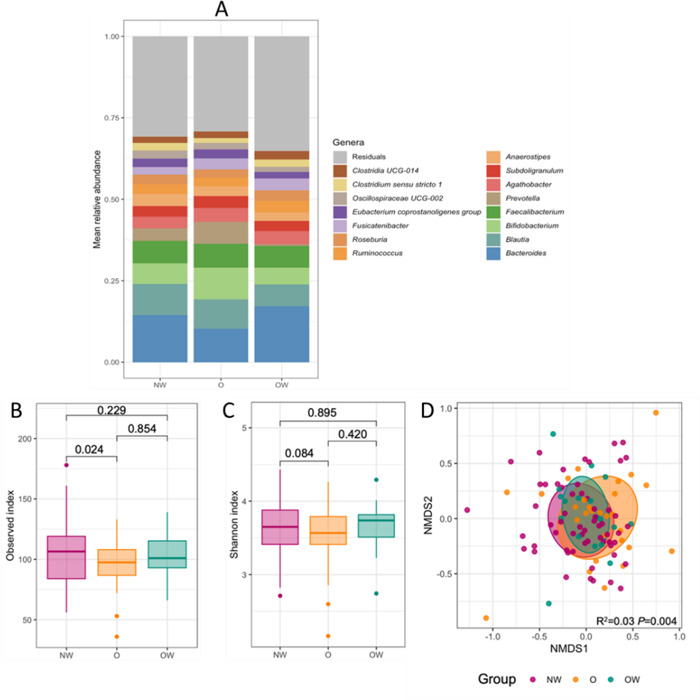
Description of the children’s gut microbiota according to the study groups assessed by 16S rRNA gene amplicon sequencing. (**A)** The figure displays the mean relative abundance of bacterial genera in the study groups. (**B and C)** Box plots of the α-diversity indices of the microbiota across the three groups. Medians and interquartile ranges are shown. Group differences in observed number of ASVs and Shannon α-diversity were calculated using multivariable linear models on the raw data and log transformed, respectively, and corrected for age. *P* values shown in the figure correspond to pairwise comparisons assessed with the multcomp package. (**D)** NMDS plot based on Bray-Curtis dissimilarity between samples, with data points and ellipses colored by study groups. Results of the PERMANOVA test (*R*^2^ and *P* value corrected for age) are indicated within the plot. Ellipses represent the standard deviation of all points within a study group.

The microbiota composition from a total of 106 samples was characterized by 16S rRNA gene amplicon sequencing that initially rendered a total of 1,297 amplicon sequence variants (ASVs). After processing the data as described in Materials and Methods, these samples resulted in 2,393,331 high-quality reads (mean ± SEM, 22,578.59 ± 841.85 reads per sample with a minimum Good’s coverage of 99.98%). These reads were binned into 433 ASVs, representing 136 taxonomic genera from six phyla. Figure 3A represents the top 15 ranked genera across the three study groups. The overall microbial community across all study groups was strongly dominated by *Bacillota* taxa, which accounted for 69.1%, followed by *Bacteroidota* and *Actinomycetota* with 20.9% and 8.4% of the relative abundance, respectively. The phyla *Pseudomonadota* (0.76%), *Verrucomicrobiota* (0.75%), and *Desulfobacterota* (0.06%) were minority taxa groups. We observed an interesting decrease in *Bacteroides* and an increase in *Bifidobacterium* in O compared to NW and OW groups. The top 20 most abundant ASVs in the overall data set were *Blautia faecis* (21), *Dorea longicatena* (22), *Ruminococcus bromii* (16), *Romboutsia* (15), *Prevotella* (18), *Subdoligranulum* (14), *Faecalibacterium prausnitzii* (19), *Subdoligranulum* (13), *Bifidobacterium adolescentis* (12), *Bifidobacterium* (11), *Prevotella* (8), *Blautia massiliensis* (10), *Anaerostipes hadrus* (7), *Bifidobacterium* (9), *Fusicatenibacter saccharivorans* (6), *Bacteroides vulgatus* (5), *Bacteroides* (4), *Faecalibacterium prausnitzii* (3), *Agathobacter* (2), and *Blautia* (1).

Species richness and Shannon’s diversity index values were significantly different between NW and O groups (Fig. 3B and C). Moreover, β-diversity showed that the microbiota structure was significantly different between the three study groups (*R*^2^ = 0.029, *P* = 0.004) (Fig. 3D).

**Fig 4:**
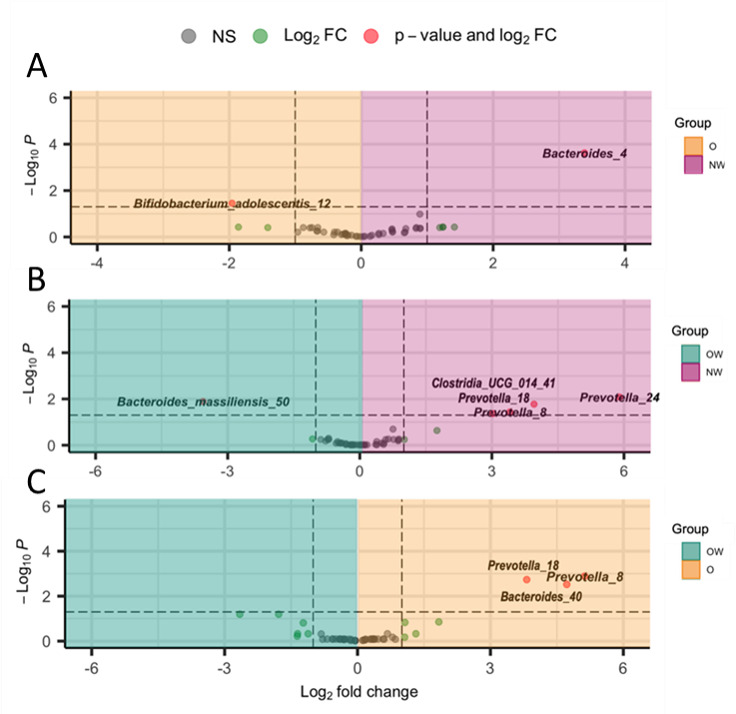
Volcano plot depicting differential abundant ASVs related to the three study groups. (**A–C)** Shade color corresponds to the study group where the specific ASVs were enriched. Gray dots represent species that were not differentially abundant; green dots represent ASVs that had a log2(FC) >1 but were not significantly differentially abundant after correcting for multiple testing; red dots represent ASVs that were significantly differentially abundant and had a log2(FC) >1. All these tests were corrected for age. Abbreviations: FC, fold change; ns, non-significant.

Significant differences have been observed in ASV relative abundances between the NW, O, and OW groups (Fig. 4) . Different ASVs belonging to species of *Prevotella* genera were significantly fluctuant and enriched in the NW or O compared to OW group. Specifically, ASVs from *Clostridia* were significantly overrepresented in NW compared to O and OW groups. *Bifidobacterium adolescentis* was significantly overrepresented in O compared to NW and OW groups. Conversely, ASVs assigned to *Bacteroides massiliensis* genera were enriched in OW group compared to NW.

### Microbial community networks according to the study groups

**Fig 5:**
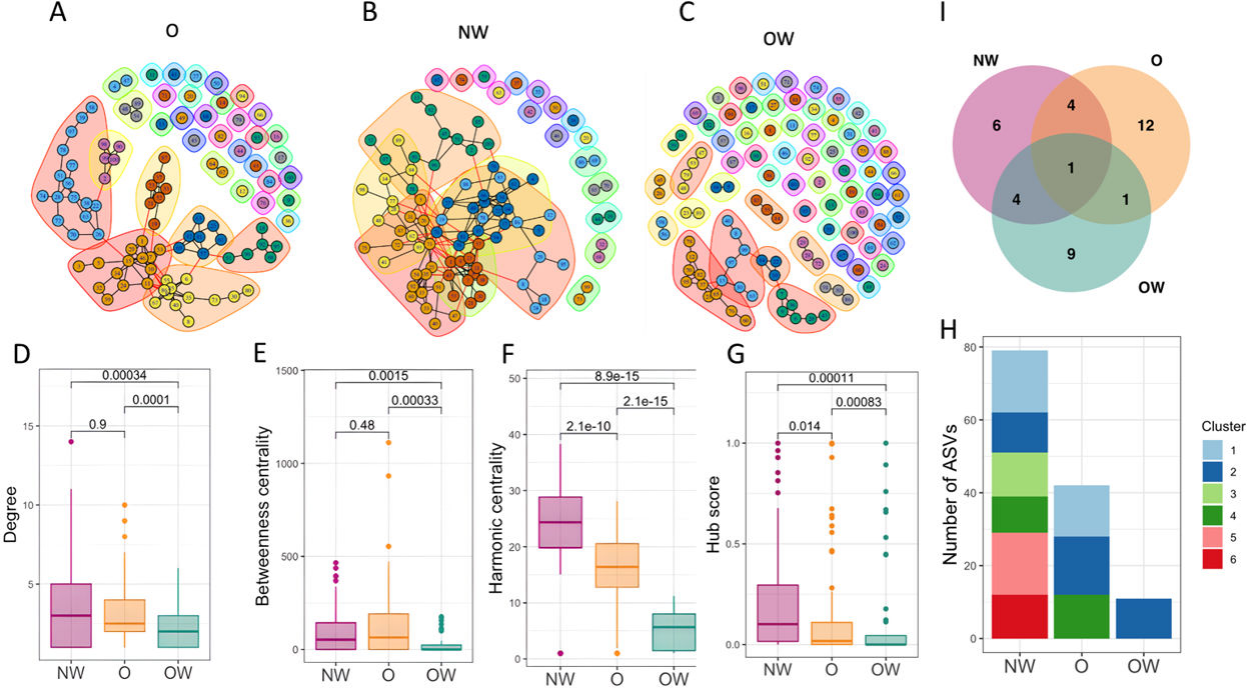
Microbial networks and network metrics for each study group. (**A–C)** Overall networks of the gut microbial community for each study group. Nodes represent individual ASVs. Shaded areas around groups of nodes represent clusters defined by cluster fast greedy community analysis. (**D–G)** Degree, betweenness, harmonic centrality, and hub score, respectively, across the three study groups. (**H)** Main clusters (clusters with more than 10 ASVs) and number of ASVs within each cluster for the three study groups networks.** (I)** Venn diagram analysis of the hub taxa shared within the study groups.

The top 100 most abundant ASVs present in at least 50% of samples in each group were selected to build a network using the SpiecEasi pipeline (Fig. 5A, B, and C). The centrality of the networks was studied by analyzing nodes’ degree, betweenness, harmonic centrality, and hub score measures for comparison purposes. Harmonic centrality is an alternative to closeness centrality for disconnected networks (25). The bacterial clusters within each network were studied, and ASVs with an important role for network structure herein as hub taxa were identified.

Overall, the ecological networks were markedly different among the study groups. The highest number of disconnected nodes was observed in the OW network followed by the O and the NW networks. The values of average node’s degree and betweenness centrality were significantly lower in OW network compared to O and NW networks (Fig. 5D and E). Harmonic centrality and hub score values differed significantly between groups with NW network showing the highest values (Fig. 5F and G). Modularity values calculated with fast greedy clustering were high for the OW network (0.75) followed by O (0.70) and NW (0.56) networks. The number of main clusters, defined as clusters with 10 or more ASVs (26), was higher in the NW network compared to the O network (Fig. 5H). Interestingly, the OW network had just one main cluster in agreement with the high number of disconnected nodes found in this network. Altogether, these results indicated a higher general community connectivity in the NW network compared to O and OW networks.

In addition, specific taxa were identified as important players in the microbial community; to demonstrate it, the degree, betweenness centrality, harmonic centrality, and hub score for each of the ASVs were calculated in each network (data not shown). The 37 hub taxa found, the group where they were identified as being important (NW, O, and/or OW), and the conditions they met were listed. One of the identified hub taxa were present in all three study groups, indicating that different ASVs are central to the network structure of the different study groups. There was some overlap regarding hub taxa between the NW and O networks (4) and between the NW and OW networks (4). However, only one common hub taxa was shared between O and OW networks (Fig. 5I).

### Interactions of BPA-tolerant bacterial community in the gut microbiota: seeking for biomarkers

The microorganisms isolated and selected through culturomics were assigned to 26 genera with tolerance to BPA (20 or 50 ppm), of which 14 were also identified by 16S rRNA gene sequencing approach considering the original data set without applying the filtering described in Materials and Methods. This highlighted that culturomics has a better potential for detecting minority taxa groups compared to metagenomics-based approach. Five of those 14 genera were present in a very low number of samples (<5 samples), namely, *Limosilactobacillus*, *Ligilactobacillus*, *Staphylococcus*, *Klebsiella*, and *Acinetobacter*. The remaining nine genera were *Bifidobacterium*, *Romboutsia*, *Intestinibacter*, *Clostridium sensu stricto 1*, *Lactobacillus*, *Turicibacter*, *Bacillus*, *Escherichia-Shigella*, and *Lacticaseibacillus* (present in ≥5 samples). These nine genera were defined as potential taxa biomarkers within the BPA-tolerant microbial community. These genera were selected for further analysis of interactions and interplay with the global gut microbiota community. The abundance of ASVs from the selected biomarker genera was generally low. However, analysis of the data revealed that the abundance of these ASVs was higher in OW and O groups than in NW group, with the exception of the *Clostridium sensu stricto one* and *Escherichia-Shigella* genera. *Enterococcus*, *Staphylococcus*, *Bacillus*, *Escherichia-Shigella*, and *Clostridium* were the genera that exhibited the highest BPA tolerance and, consequently, were the most abundant genera identified by culturomics. This result partially contrasted with 16S rRNA gene amplicon sequencing data where only ASVs assigned to *Escherichia-Shigella* and *Clostridium* genera were highly abundant. ASVs assigned to *Enterococcus*, *Staphylococcus*, and *Bacillus* were present in few samples with low relative abundances.

### Cultured taxa biomarkers impacting the dynamics of overall microbial community: α- and β-diversity indices

**Fig 6:**
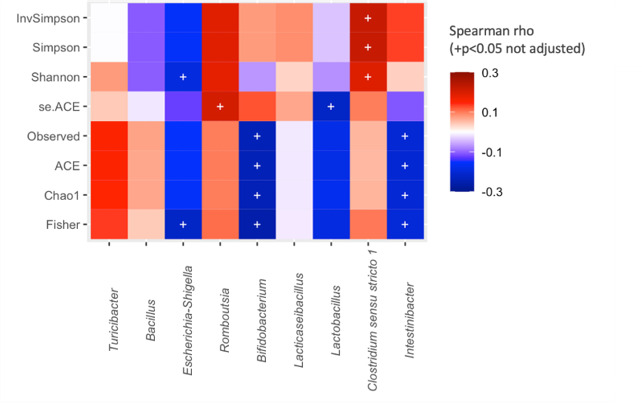
Cultured taxa biomarkers’ influence on the α-diversity indices of the microbial community. Spearman’s rank test shows the correlation between discriminant taxa of BPA tolerance with α-diversity metrics.

Next, the correlation between the relative abundance of BPA-tolerant biomarker genera with the α-diversity indices was calculated based on the 16S amplicon sequencing data. *Clostridium sensu stricto 1* and *Romboutsia* were positively correlated with diversity, according to Shannon, Simpson, and ACE indices. *Intestinibacter*, *Escherichia-Shigella*, *Bifidobacterium*, and *Lactobacillus* were inversely correlated with diversity according to observed, ACE, Chao, and Fisher indices (Fig. 6).

**Fig 7:**
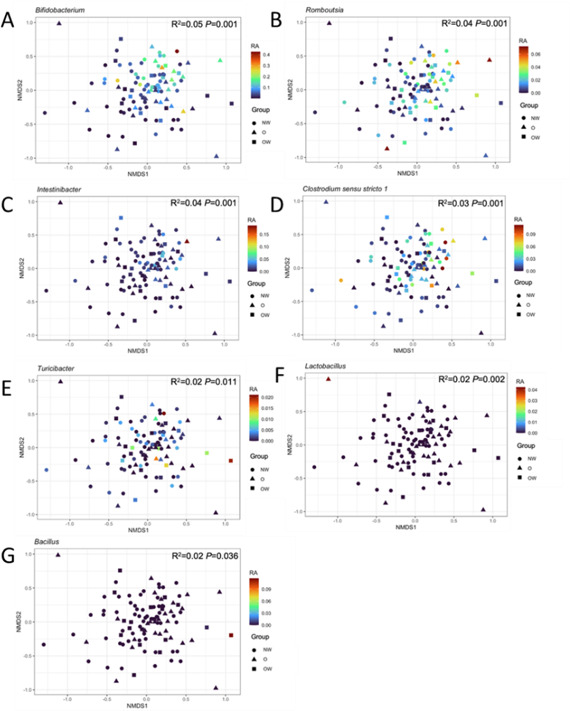
Correlation between biomarker’s genera and community structure based on β-diversity. NMDS plot based on Bray-Curtis dissimilarity between samples, with data points shaped by study groups and colored by relative abundance of the biomarker’s genera. (**A)** *Bifidobacterium*; (**B)** *Romboutsia*; (**C)** *Intestinibacter*; (**D)** *Clostridium sensu stricto* 1; (**E)** *Turicibacter*; (**F)** *Lactobacillus*; (**G)** *Bacillus*; results of the PERMANOVA test (*R*^2^ and *P* value) are indicated within the plots.

The correlation between biomarker genera and β-diversity was performed. *Bifidobacterium*, *Romboutsia*, *Intestinibacter*, *Clostridium sensu stricto 1*, *Lactobacillus*, *Turicibacter*, and *Bacillus* showed significant effects (*P* < 0.05) on microbial community structure (Fig. 7). *Bifidobacterium*, *Romboutsia*, and *Clostridium sensu stricto 1* had the highest *R*^2^ values in the β-diversity analysis, indicating a strong influence on microbial community structure. *Escherichia-Shigella* and *Lacticaseibacillus* genera did not show any significant correlation with β-diversity values.

### Cultured taxa biomarkers impacting the microbial community networks

**Fig 8:**
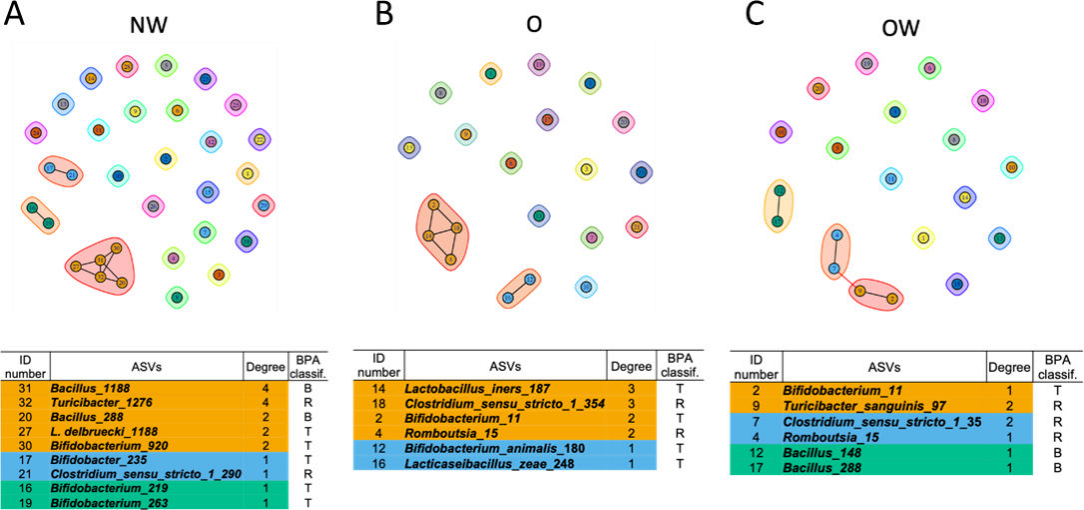
Microbial subnetworks of biomarkers ASVs for each study group. (**A–C)** Subnetworks of the BPA-tolerant gut microbial ASVs for each study group. Nodes represent individual ASVs. Shaded areas around groups of nodes represent clusters defined by cluster fast greedy community analysis. BPA metabolic classification: B, biodegrader; T, tolerant BPA; R, resistant.

The purpose of the network analysis was to identify potential species interactions within the experimental groups. The combination of BPA-tolerant selected biomarkers with 16S rRNA gene amplicon sequencing characterization allowed us to elucidate cooperative or antagonistic relationships between specific species (Fig. 8). These species potentially degrade BPA through pathways according to their theoretical prediction of gene encoding enzymatic arsenal. Previous studies described the isolation, identification, and description of BPA biodegradation capacities: BPA-high biodegrader, BPA-medium biodegrader or tolerant, or BPA-resistant taxa according to their genes encoding enzymes belonging to the four BPA pathways (>40%, >19%, <19%, and >12%, respectively), allowing to establish theoretical and differential BPA biodegradation capacities as described above (21). The microbial association profiles found regarding BPA degradation were added to the subnetworks constructed from the AVSs belonging to the biomarker’s genera selected. The BPA degradation profiles were different according to the study groups (Fig. 8).

The better connected ASVs were found in the NW subnetwork, with nodes linked by four or two edges. Within this subnetwork, the following patterns according to their association and potential BPA enzymatic capacities were observed: in NW group, (i) biodegrader-resistant-tolerant-biodegrader taxa pattern, represented by members of the genera *Bacillus-Turicibacter* (biodegrader-resistant), *Bacillus-Lactobacillus*, or *Bacillus-Bifidobacterium* (biodegrader-tolerant); (ii) tandem of tolerant-resistant (*Bifidobacterium-Clostridium sensu stricto*); (iii) tandem of tolerant-tolerant (*Bifidobacterium-Bifidobacterium*) (Fig. 8A). NW subnetwork possesses a better connectivity and diversity taxa including bacteria with high enzymatic potential in BPA degradation together with medium or low BPA degradation bacteria. In the O subnetwork, we only found tolerant-resistant (*Lactobacillus-Clostridium sensu stricto* or *Bifidobacterium-Rombutsia*) and tolerant-tolerant (*Bifidobacterium-Lactobacillus*) tandems (Fig. 8B). None of the patterns observed in the O subnetworks were connected to ASVs with potential BPA biodegrader capacities. In addition, differential tandem connections were present in the OW subnetwork: (i) biodegrader-biodegrader (*Bacillus-Bacillus*), (ii) tolerant-resistant (*Bifidobacterium-Rombutsia*), and (iii) resistant-resistant (*Clostridium sensu stricto-Turicibacter*) (Fig. 8C). There were more ASVs belonging to BPA-cultured genera distributed within the NW subnetwork (27) compared to the O (21) and OW (20) groups.

## DISCUSSION

Cumulative exposure to xenobiotics and obesity seem to be linked as key risk factors for chronic noncommunicable diseases, which are the number one cause of death and disability in the world (28, 29). Specifically, efforts for understanding the triggering factors and mechanisms involved in the development of childhood obesity are highly recommended to face this worldwide health problem (30). Recent evidence highlights the crucial role of the gut microbiota in maintaining health status, with microbial imbalances potentially leading to specific diseases (31). Nowadays, humans are exposed to numerous xenobiotics as a result of diet and daily life activities, such as pesticides, antibiotics, parabens, phthalates, and bisphenols; many of them have resulted in demonstrated inflammatory and obesogenic effects (32). Recent studies have indicated that apart from human enzymes, the gut microbiome also contributes to metabolizing xenobiotics. However, the interaction between gut microbiome and biotransformation of environmental contaminants is very complex and not yet fully understood (27). To discover the drivers, associated biomarkers, and potential biotools for modulating the effects of xenobiotics on gut microbiota and, ultimately, for influencing human health, it is necessary to adopt more holistic and integrative omics approaches (33). Culturomics- and metagenomics-based approaches can be combined to provide a better understanding of microbiological communities (34). Here, we observed that not all the genera isolated by culturomics were present in the 16S rRNA gene amplicon sequencing results, in agreement with previous studies showing that culturomics can detect minority taxa that might be underrepresented by using metagenomics approaches (23, 35, 36). Additionally, culturomics partially fills gaps in 16S rRNA gene amplicon sequencing approaches by identifying particular sequences that were previously categorized as non-cultivable or unassigned in earlier studies (24). In our study, we were able to assign to the species level all the isolates, while the amplicon sequencing approach only achieved genus level in 66.7% of the ASVs described. However, culturomics has a big limitation known as “the great plate count anomaly.” Gradually, the number of uncultured bacteria will decrease as we improve the culture conditions (23). We included numerous isolation media and conditions (including anaerobiosis) to address this issue, increasing the rate of positive bacterial isolates. We have noted that exposure to BPA together with culturing approach, media, conditions, and picking colony methodology could insert an unavoidable bias in the selection of cultivable species. It is also well known that several species from *Bacillota* and *Bacteroidota* were differentially sensitive to the BPA, e.g., *Lactobacillus* and *Bacteroides* were lost after BPA, and meanwhile, sporobiota (*Bacillus* and *Clostridium*) and *Prevotella* remain, respectively. In any case, culturomics- and metagenomics-based approaches are complementary and necessary for the gut microbiota study and overcome these issues (37, 38).

BPA exposure of 3 days was enough to isolate differential taxa from the samples. Previous studies have shown that *Bacilli* taxon is the most prevalent Gram-positive bacteria capable of degrading BPA (39). Additionally, potentially probiotic strains from the genera *Lactococcus*, *Bacillus*, *Lactobacillus*, and *Enterococcus* have been tested for their tolerance to BPA toxicity and their ability to remove it (40). These results are in line with our study findings, where *Enterococcus* and *Bacillus* were the predominant genera isolated in the presence of BPA. Among the genera that were more frequently isolated, we also obtained *Staphylococcus*, *Escherichia-Shigella*, and *Clostridium*, indicating a possible direct effect of selective pressure by the BPA. These three genera have been classified as intermediate xenobiotics metabolizers in a previous study (27) and presumably may be considered as drivers of the xenobiotic-metabolizing enzyme repertoire in a gut microbiome. Interestingly, several microbiota predominant genera, involved in homeostasis, immunity, and obesity, such as *Bacteroides*, *Prevotella*, *Oscillospira*, and *Bifidobacterium*, were not isolated from any of the study groups, but they were identified as main fluctuant taxa by 16S rRNA metagenomic between groups. It is possible that these taxa may be potentially affected by the presence of bisphenol xenobiotics, decreasing *Bacteroides* and *Prevotella* (41), or that culture conditions do not cover all culturing requirements (e.g., enriched and anaerobic conditions for *Bifidobacterium* growth (42). We found the highest unique number of species in the NW group, compared to O and OW, which could contribute to a more efficient BPA degradation.

16S rRNA gene amplicon sequencing profiling revealed significant changes in the overall microbiota structure but not in species richness associated to obesity, as it has been observed in previous studies (43–46). However, differences regarding the *Firmicutes*/*Bacteroidetes* (F/B) ratio and individual taxa among children with overweight/obesity compared to normal-weight children are more controversial in literature (47, 48). In our study, no statistical differences were found in F/B ratio between the groups, in line with some studies where no associations were found between this ratio and the BMI (49). Regarding individual taxa associated to obesity, for instance, Hollister et al. found that *Bacteroides massiliensis* and *Bacteroides plebeius* were enriched in the stool of children with obesity (46), and in our study cohort, *Bacteroides massiliensis* was significantly overrepresented in the overweight group. We also observed that *Bifidobacterium adolescentis* was associated with obesity in children; nevertheless, this genera showed lack of consensus regarding obesity (48). Especially interesting is the association of the genera *Prevotella* with obesity, of which we found several ASVs significantly increased in the normal-weight and obesity groups. In spite of previous studies reporting an increase in this genera in the gut microbiota of individuals with obesity (50–52), other independent studies showed that individuals with the *Prevotella*-driven enterotype appeared more susceptible to lose body fat on fiber-rich diet (53, 54). Our results and previous studies suggest that the relationship between obesity, nutrition, and the microbiota is complex and multifactorial (55). While the link between the microbiota and obesogenesis is unclear, research suggests that microbial composition can be influenced by diet and is associated with body weight and obesity.

Network analyses were performed to gain a better understanding of the complexity of microbial interactions operating within each of the study group samples. The bacterial community networks showed significant differences; specifically, we found that the normal-weight network had a higher overall community connectivity, represented by a lower number of disconnected nodes, higher average harmonic centrality, and average hub score compared to the obese and overweight networks. Dugas et al. also found lower network connectivity in the gut microbiota of individuals with obesity compared to normal-weight individuals in a population of Ghanaians women (56). Cho in 2021 (57), using a network approach, found that in obese children who followed a weight reduction program, the group of children that gained fat after the intervention presented a higher degree of dysbiosis. In soil environments, networks less complex, with lower connectivity and lower number of clusters that consist of less nodes, were associated with plant diseases. On the contrary, networks that were better organized and contained more interacting species were associated with health-related stages (58). Besides, complex networks with high connectivity are more resilient to environmental stressors than simple networks with limited interactions (59, 60). We can speculate that the higher complexity of the normal-weight microbial network structure may suggest that the members of this community could complement each other to withstand environmental perturbations such as xenobiotic exposure, abiotic stress, or pathogen attack. However, this finding needs further verification with functional analysis, ideally combining multiple omics approaches (to see how it is related to development of obesity and xenobiotic degradation) to make a valid conclusion on this hypothesis (61).

After defining the selected genera as potential biomarkers in relation to BPA tolerance, our data analysis revealed that their abundance was higher in OW and O groups compared to the NW group. Previous studies have associated a gut microbiota enriched in *Lactobacillus* (62, 63), *Intestinibacter*, and *Romboutsia* (64) with obesity phenotype. Prior research has shown that normal-weight children have a higher diversity of microbiota compared to children with obesity (65). Therefore, we hypothesized that selected biomarkers that were positively correlated with diversity would be overrepresented in the normal-weight group, while those that were negatively correlated with diversity would be overrepresented in the obesity group. We found that *Clostridium* and *Turicibacter* positively correlated with diversity and were overrepresented in the NW group. In contrast, *Intestinibacter*, *Bifidobacterium*, and *Lactobacillus* inversely correlated with diversity and were overrepresented in the O group. Several studies have reported that BPA exposure changes microbial community structure in favor of some genera like *Lactobacillus*, *Acidovorax*, *Stenotrophomonas*, *Megasphaera*, *Microbacterium*, and *Alcaligenes* (66, 67), triggering an imbalance of the community diversity. Some of the selected biomarkers have been tested for its versatility for BPA or xenobiotic degradation, such as *Lactobacillus* that was able to degrade almost 70% of BPA added to the medium (40, 68) and *Intestinibacter* and *Bifidobacterium* that were classified as intermediate versatility according to the overall xenobiotic degradation (27). However, *Clostridium* was classified as having low versatility in metabolizing xenobiotics, and there are no data on *Turicibacter*’s potential to degrade BPA (27) further than being involved in lipids and bile acid metabolisms (69). This suggests that BPA exposure could contribute to the establishment of differential microbial communities in children with obesity and normal weight. More efforts should be done to determine which species are selected by xenobiotics that trigger obesity phenotypes. Further investigation is needed to establish a connection between the promotion of the overall microbial diversity from specific taxa, obesity, and BPA exposure of the samples. The controversial outcome of several genus, such as *Romboutsia* (70) and *Escherichia-Shigella* (71), could be due to their diverse functionalities, complex taxonomy, and the need to better identify the species and strains involved in gut eubiosis-dysbiosis balance.

It is also relevant to highlight the decisive role of *Bifidobacterium* as a dominant and symbiotic inhabitant of the human gut due to its capacity to cope with a multitude of chemical, oxidative, osmotic, and bile salt/acid stresses (72) together with its wide metabolic capacity and versatility for degrading aromatic amino acids (73). Following this premise, *Bifidobacterium* taxa could be resistant to the presence of BPA, metabolizing it and consequently driving community changes. In any case, the abundance of specific species and strains has led toward both obesogenic (62) and antiobesogenic effects (74).

The individual’s xenobiotic metabolism or detoxification capabilities are determined by the abundance and variety of enzymes in their microbiome, which can decrease the bioavailability of specific xenobiotics. However, there is limited information available on specific clades of bacteria and their classification based on their xenobiotic-metabolizing capacities (27). In our study, plausible complete BPA degradation by the gut microbiota should be attributed to several microbial community taxa instead of single species. This hypothesis enhances the putative role of the subnetworks found, where the better-connected taxa and more diverse and more efficient metabolic associations were depicted in the NW group. Importantly, putative BPA-degrader components such as *Bacillus* spp. showed a better distribution for favoring collaborative metabolism or degradation in NW individuals with intermediate- or non-degraders taxa, compared to simpler and homologous patterns established in OW and absent in O group. Therefore, these taxa may be considered as a BPA-metabolizing driver. It is also relevant to highlight that the cumulative presence of intermediate metabolites with different toxigenic degrees could chronically increase specific xenobiotic metabolizers affecting the dynamics of the community and host physiology (75). In any case, keystone taxa found in our study could become a microbial resource for use in further interventional studies, as recognized safe microorganisms as single or multiple probiotics (76, 77).

Limitations of our study are mainly inherent to the experimental method, specifically, 16S rRNA gene sequencing, which can limit the resolution of the taxonomy that can be assigned to the bacteria species. However, combining these results with culturing, we could describe the BPA- resistant taxa at the species level and their role at the gut community dynamics. In addition, future functional analysis, such as metatranscriptomics, metaproteomics, and metabolomics, will allow to better understand the biological mechanisms underpinning these findings. We performed a theoretical functional description of the subnetwoks based on BPA degradation capability prediction already described in literature. Still, further *in vitro* and *in vivo* studies are needed to verify the role of the BPA-tolerant taxa in the gut community and its influence in obesity and to ultimately describe and test bacterial isolates that could potentially be used as probiotics to counteract the obesogenic effect of certain xenobiotics.

This study investigates the influence of BPA-tolerant genera on gut microbial ecology and whether these taxa could serve as potential indicators or drivers to trace the effects of BPA exposure on microbiota composition and dynamics and whether these effects could be linked to obesogenic outcomes. Moreover, combined culturing and metagenomics data analysis allowed to identify the specific taxa impacting the diversity indices of the gut microbial samples. These data support the hypothesis that there are BPA microbiota drivers (either single or in consortia) within the community, which are capable of establishing patterns of lower or higher diversity, which in turn define obesogenic or antiobesogenic phenotypes, respectively. The strength of microbiota culturing methods will allow us to obtain biotools for further uses as biotic resources (probiotics, enzymes, prebiotics, and postbiotics). Therefore, we have demonstrated that integrating complementary culturing and omics data can enhance our understanding and provide more holistic scientific evidence of potential and biotechnological biomarkers for obesity. This study, for the first time, proposes potential cultured microbiota biomarkers linked to the BPA xenobiotic exposure and obesogenic phenotypes. Moreover, taxa of interest could be further studied and proposed for BPA degradation or as bioremediation tools in several areas under the One Health concept.

## MATERIAL AND METHODS

### Participant characteristics

A total of 106 children, 55 boys (50.9%) and 51 girls (47.2%), with a median age of 7.7 ± 2.5 years were enrolled. Anthropometric characteristics are summarized in Table 1. The anthropometric classification was performed according to guidelines from WHO 2007 (78) in NW (*n* = 60), OW (*n* = 18), and O (*n* = 30). Participants were selected from a panel of children who took part in the OBEMIRISK study (79), a cohort study of children with a permanent address in Granada, Spain. The participants did not have any intestinal disorders and had not taken antibiotics within the previous 3 months. Anthropometric measurements and lifestyle questionnaires were collected according to the study guideline. Between September 2020 and April 2021, all fecal samples were collected using anaerobic kits and then immediately frozen at −20°C. The samples were stored at −80°C until further processing. Study permission was obtained from the Institutional Ethic Committee from the University of Granada (CEIM/CEI 23/12/2019-Acta12/19), and all the work was carried out in compliance with the Declaration of Helsinki. The study was explained to the participants before enrollment, and written informed consent was obtained from each child’s legal guardian.

**TABLE 1:** Anthropometric characteristics of participants

	NW (*n* = 60)	O (*n* = 28)	OW (*n* = 18)	*P* value
Gender				0.196
Female	28 (46.7%)	11 (39.3%)	12 (66.7%)	
Male	32 (53.3%)	17 (60.7%)	6 (33.3%)	
Age (year)				<0.001
Mean (SD)	6.917 (2.499)	8.571 (1.731)	9.167 (2.256)	
Range	3.000–13.000	5.000–12.000	4.000–13.000	
BMI				<0.001
Mean (SD)	15.534 (1.378)	24.831 (3.045)	20.349 (2.297)	
Range	12.960–18.500	19.470–30.580	17.100–23.940	
Fat (%)				<0.001
Mean (SD)	19.536 (3.190)	35.337 (5.153)	28.222 (5.569)	
Range	12.200–27.500	25.100–45.500	21.900–40.500	
Muscle (%)				<0.001
Mean (SD)	75.917 (2.986)	61.163 (4.850)	64.950 (12.350)	
Range	68.600–83.400	51.700–70.900	19.800–73.300	
Waist (cm)				<0.001
Mean (SD)	58.546 (5.817)	81.822 (9.734)	70.994 (11.747)	
Range	50.000–74.000	63.200–97.000	56.000–96.000	

### Culturomics—species isolation, identification, and selection

A 0.5 g of 69 fecal samples (NW [*n* = 37], OW [*n* = 11], and O [*n* = 21]) were suspended in 5 mL of Luria-Bertani media and exposed to different concentrations of BPA (0.5, 10, 20, 50, and 100 ppm) for 72 h at 37°C in anaerobic conditions with anaerobic jars through Anaerocult A system (Merck, Darmstadt, Germany), according to previous primary searching and screening studies for obtaining microbial BPA biodegrader species (80). The suspensions were serially diluted until 10^−4^, and aliquots of 100 µL of each diluted suspension were plated on six different solid media BHI, MRS, RCM, GAMa, or GAMg. The plates were incubated for 72 h at 37°C in anaerobic conditions. A total of five culture media and 25 conditions were used in this study. The experiment was initially designed to select and observe the impact of high—even overdose—levels of BPA on the resistant and tolerant gut taxa microbiota. A pre-assay with several BPA concentrations from 10, 20, 50, 100, and 200 ppm was done, and it allows a variety and number of colonies isolated and observed. However, after comparing the results of preliminary experiments, plates with 20 and 50 ppm were chosen as the best BPA culture conditions for isolating the highest number of microbial species. Samples exposed to less than 20 ppm showed too many colonies, meaning no inhibitory effects were achieved but making isolation challenging. Conversely, samples exposed to concentrations higher than 50 ppm, e.g., 100 ppm, showed high toxicity, and no colonies were isolated. In order to ensure the purity of the single colonies, two rounds of streaking on the original isolation media were performed.

Genomic DNA (gDNA) from each pure isolated culture was extracted using DNeasy columns (Qiagen, Hilden, Germany) following the manufacturer’s instructions. The gDNA preparations were used for PCR amplification using the universal primers for 16S rRNA gene: 16F27 (5′-AGAGTTTGATCMTGGCTC-3′) and 1525R (5′-AAGGAGGTGATCCAGCC-3′). PCRs were conducted using a thermal cycler (Applied Biosystems, Inc., Foster City, CA) by applying the following conditions: initial denaturation at 94°C for 5 min; 35 cycles of denaturation at 94°C for 45 s, annealing at 55°C for 1 min, and extension at 72°C for 1 min 40 s, followed by a final extension at 72°C for 10 min.

Amplified PCR products were verified on 0.8% agarose gel electrophoresis before Sanger sequencing (Institute of Parasitology and Biomedicine “López-Neyra” Service, Spain) using three universal primers for sequencing 16S rRNA gene: F357 (5′-CTCCTACGGGAGGCAGCA-3′), R519 (5′-GWATTACCGCGGCKGCTG-3′), and F915 (5′-GGGCCCGCACAAGCGGTGG-3′). 16S rRNA gene sequences were compared through BLASTn (NCBI) against published 16S rRNA gene sequences in the GenBank database. Assigned species taxonomy was accepted at a similarity level higher than 97%.

### 16S rRNA gene amplicon-sequencing and data processing

DNA extraction from stools was performed using the PowerSoil DNA Isolation Kit (Qiagen, Hilden, Germany) following the manufacturer’s instructions. The V4 hypervariable region of the 16S rRNA gene was amplified in a two-step process: first using the 515F (5′-GTGCCAGCMGCCGCGGTAA-3′) and 806R (5′-GGACTACHVGGGTWTCTAAT-3′) universal primers, and second, the specific Illumina multiplexing sequencing and index primers. The library was prepared by pooling equimolar ratios of amplicons and sequenced using an Illumina MiSeq platform. Amplification, library preparation, and sequencing were performed at RTL Genomics (Lubbock, TX). Raw sequence data were processed to obtain ASVs. First, primers sequences were removed with cutadapt (v3.4) (81). Using DADA2 (v1.16.0) (82), paired-end sequences were filtered and trimmed with the following parameters (maxEE = 2, 7; truncLen = 270, 200 bp; truncQ = 2), denoised, and merged (minOverlap = 170, maxMismatch = 0), and a sequence table was constructed. Chimeras were identified and removed (method = “consensus”). ASVs were annotated up to the genus level using the DADA2 implementation of the naïve Bayesian classifier based on the Silva v138.1 reference database (83). Species-level annotations were added using the addSpecies function. DADA2 was implemented using RStudio (v4.0.4). DADA2 output was combined into a single object using the phyloseq R-package (84), which was used for downstream analyses. ASVs that were present at a confident level of detection were selected, i.e., representing at least 0.1% of all reads in at least two samples (85). ASVs not assigned to bacteria or annotated as mitochondria or chloroplast were removed.

### Statistical analyses

Statistical analyses were carried out using R version 4.2.2 (R Core Team) within RStudio v1.2, primarily with the packages vegan (86), phyloseq (84), metagenomeSeq (87), microbiome (88), and ggplot2 (89). For continuous nonparametric variables, the Kruskal-Wallis test followed by pairwise Mann-Whitney post hoc test corrected for multiple comparisons were used to compare more than two groups. For categorical variables, the Fisher exact test was used. Benjamini-Hochberg adjusted *P* values (q values) were generated when appropriate. A *P* value and an adjusted *P* value of 0.05 were considered significant, unless otherwise stated.

For the description of the children’s gut microbial community, culturomics and 16S rRNA gene sequencing were combined, and the following analyses were performed: (i) α-diversity was estimated by observed number of taxa and by Shannon diversity index. Group differences in observed number of ASVs and Shannon α-diversity were calculated using multivariable linear models, and pairwise comparisons were assessed with the multcomp package. (ii) A nonmetric multidimensional scaling (NMDS) plot based on the Bray-Curtis dissimilarity matrix was used to visualize differences in the overall microbial community composition between study groups. PERMANOVA test was performed using the adonis2 function (vegan package) to test for the significance of stratification of the children according to BMI on the overall microbiota composition, and (iii) significant differences at relative abundances were tested (only for 16S rRNA gene sequencing results) between our study groups using the metagenomeSeq package. All these tests (α-diversity, NMDS, and volcano plots) were corrected for age.

For the investigation of the community dynamics in relation to tolerant genera, the following analyses were executed [without executing the filtering described in the previous section (32) in order to capture the information of the minority taxa). (i) A Spearman’s rank correlation was used to determine any correlations between α-diversity indices and BPA-biomarker genera relative abundance, and the heat function from the microbiome R package was used for visualization. We calculated raw and adjusted *P* values associated to the correlation. (ii) The significant contribution of the BPA-biomarker genera to the microbial community structure was assessed using the PERMANOVA test, and (iii) the bacterial interactions within the entire community and between the ASV belonging to the BPA-biomarker genera was studied by network analysis using the SpiecEasi package. For the complete community networks, only the top 100 relative abundance ranked ASVs that were present in at least 50% of the samples within each group were considered for the analysis. To infer bacterial interactions within the spiec.easi function, the method glasso was used, with lambda.min.ratio = 1e-2 and nlambda = 40. The igraph package was used for visualization and network metrics calculation. The modularity was investigated within each network using the cluster fast greedy algorithm. To identify hub species (i.e., ASVs playing a significant role in determining the structure of a community network), four important network metrics for each ASV were calculated within each network. Hub species were defined as those ASVs with high degree (the number of connections each ASV has within a network), high betweenness centrality (number of shortest paths that pass through a specific ASV within a network), high harmonic centrality (a measure of closeness centrality extended to unconnected graphs), and/or high hub score (a measure of how connected the node is to other influential nodes) (90, 91). The ASVs at the top 10% of the distribution of each of those metrics were selected. ASVs within each study group that met at least one of those conditions were considered hub taxa.

## Data Availability

Culturing 16S rRNA gene from isolates-Sanger sequences have been deposited in NCBI GenBank (OR078042–OR078374), and 16S rRNA gene V4-amplicon metagenomic sequencing reads and metadata have been deposited in NCBI Sequence Read Archive (SRA) database with the Bioproject number PRJNA979040.
